# 

**Published:** 1911-10-21

**Authors:** 


### Gifts.

The borough member (Viscount Lewisham) has become an annual subscriber of £25 to the West Bromwich District Hospital, and an additional £50 (making £1,336 in all) has been received from the executors of the late Mr. William Marsh.

The late Mr. Isaac Lovell Berridge, of Leicester, has bequeathed £500 to the Leicester Infirmary, subject to the life interest of Mrs. Berridge.

Mrs. and Miss Morley, of Groby, as the result of a sale of work at Branting Hill, have been able to send fifty guineas to the Leicester Infirmary, and we understand that the name of Mrs. Morley is to be submitted to the governors for election to a life-governorship.

A large sum, probably about £75,000, has been left by Mr. Edward Davis, of Cheltenham, for the benefit of such general hospitals or infirmaries in Scotland as may be selected by the trustees.

Mr. William Arthur Daubeney, of Dawlish, has left £100 to the Bristol Royal Infirmary.

Mr. H. D. Hamilton Ferg'son, of Eaglefield Green, has bequeathed £100 to the Egham and Eaglefield Green Cottage Hospital.

The Malvern Rural Hospital will receive £200 under the will of the late Miss Jane Clarke, of Great Malvern.

Mr. Wm. Henry Wellstead, of Falmouth and Reading, has left a considerable bequest to the Royal Berkshire Hospital, Reading.

By her will the late Mrs. Susannah Love, of East Parade, Harrogate, directed that the residue of her estate should be divided as to one-tenth to the Bradford Royal Infirmary; one-tenth to the Huddersfield Infirmary; one-tenth to the Yorkshire Home for Incurables, Harrogate; and one-tenth to be divided equally between the Harrogate Cottage Hospital and the Royal Bath Hospital, Harrogate. The total amount for division among the residuary legatees is expected to be £11,260.

Mrs. Rachel Feldheim has left £100 to the Brompton Hospital for Consumption and £100 to the Jewish Home for Incurables.

Mr. Albert Wood has given £12,500 towards the extension scheme of the Chester Infirmary. This gift will be applied to the erection of two new wards, which will be named after the benefactor.

The Chester Infirmary has also received a gift of £1,000 from Miss Best, of Cleveland Square, W., for the endowment of a bed in memory of her sister, formerly a resident at Chester.

Mrs. Louisa Ashton has bequeathed £200 to the Blackburn and North East Lancashire Infirmary.

Mr. Gibson, a former Mayor of Newcastle, has left £2,000 to the Newcastle Royal Infirmary.

Miss Ellen Baker has left £500 to the Worthing Infirmary.

Colonel Wm. A. Pennington has bequeathed £200 to the Royal South Hants Hospital, Southampton, and £100 to the Warneford Hospital, Leamington.

Under the will of the late Mr. Major Mellor, the University College Hospital receives £105, and the following hospitals £100 each: St. Mark's Hospital for Fistula, the Middlesex Hospital for Cancer Research, and the London Hospital.

Miss Jane Pittis How has left £100 each to the Royal Isle of Wight Infirmary and the Royal National Hospital for Diseases of the Chest.

Mr. Robert Sheil, of Dublin, has left £100 each to Sir Patrick Dun's Hospital and to the Royal City of Dublin Hospital.

Mr. Edward Paine Rose, of Bedford, has left £1,000 to the Bedford County Hospital.

Mr. Henry Nathan has left, subject to the life-interest of his wife, £500 to the Hospital for Incurable Children at Hampstead and £500 to King Edward's Hospital Fund.

Sir Chandra Shum Shere Jung has given £1,000 towards the new laboratory extension fund at the Brompton Hospital for Consumption.

INSTITUTIONAL WORKERS:

Are you seeking an appointment? A large number of vacancies are announced in this week's issue of The Hospital. The Hospital is on sale at all newsagents and bookstalls throughout the country.

### Buildings Contemplated and in Progress.

City of London.—The Finance Committee have recommended to the Board of Bow that certain renovations to the Infirmary be carried out at a cost of about £10,000. The recommendation was adopted. Mr. A. E. Pridmore is the architect.

Lickey (Worcestershire).—Messrs. Martin and Martin, of Birmingham, are architects for the sanatorium, to cost £7,000.

Newcastle-on-Tyne.—A new pavilion has been added to the Home for Incurables, containing on the ground floor a girls' ward for twelve beds, and on the first floor a similar ward for boys. The second floor contains eleven staff bedrooms. Messrs. Shewbrooks and Hodges, of Newcastle, are the architects.

Plymouth.—Additions are contemplated to the Throat and Ear Hospital at a cost of £2,000. Messrs. Thorneley and Rooke, of Plymouth, are the architects in charge.

Romford.—Alderman Banks-Martin, J.P., of East Ham, has generously offered his services free as architect for the proposed enlargement of Romford Hospital, which is to be known as a King Edward Memorial. His services have been accepted, and he estimates the cost at about £1,200.

Skegness.—E. R. Sutton, Esq., F.R.I.B.A., has given an award in connection with the architectural competition for Skegness Cottage Hospital. Mr. F. J. Parkinson, of Blackburn, is the winner; fifty-six sets of drawings were submitted.

Tewkesbury.—Tenders are invited for alterations and additions to the Isolation Hospital at Wedington. Approved sureties will be required. The plans may be seen at the office of the architect, Mr. James Villar, M.S.A., F.S.I., 5 Essex Place, Cheltenham. An inquiry by the Local Government Board for powers to borrow £5,000 for the scheme was held several weeks ago.

Note.—Dr. Mackintosh, M.V.O., was consulted about the buildings at Arbroath and Kilmarnock referred to in this column last week.

### Appointments.

Mr. J. Falconer Brown, M.B., Ch.B., of Edinburgh, has been appointed house physician to the Leicester Infirmary. He graduated with first-class honours in July 1910, and for the past fourteen months has been house surgeon to the Bradford Royal Infirmary.

Mr. C. F. Walker, M.D. Lond. (State Med.), B.S. Lond., D.P.H. Vict., M.R.C.S. Eng., L.R.C.P. Lond., B.A., R.U.I., has been appointed resident medical officer of the Leicester Isolation Hospital. Mr. Walker was educated at Belfast and St. Mary's Hospital, London, and has held the post of senior resident at the South Devon and East Cornwall Hospital, Plymouth.

Mr. Keith B. MacGlashan, M.B., Ch.B. Edin., and Dr. H. S. Metcalfe, B.A., M.D., B.Ch., B.A.O. Ireland, have been appointed house-surgeons to the Bradford Royal Infirmary.

At a meeting of the Special Election Committee held on October 6, Mr. Charles Lupton in the chair, George W. Watson, M.D., was elected a member of the honorary staff of the General Infirmary at Leeds.

Mr. Frederick C. Pybus, M.S. Durh., F.R.C.S., has been appointed Assistant Surgeon to the Hospital for Sick Children, Newcastle-on-Tyne.

Mr. Sydenham F. Moore, M.B., B.Ch. Oxon., M.R.C.S. and L.R.C.P., has been appointed House Surgeon to the Warneford, Leamington and South Warwickshire General Hospital, Leamington Spa.

Mr. William Messer, M.B., Ch.B. Edin., has been appointed House Physician to the Bradford Royal Infirmary.

Mr. Wilson H. Hey, M.B., F.R.C.S.E., has been appointed a Visiting Surgeon to the Manchester Children's Hospital, Pendlebury, near Manchester.

Out of a list of eleven candidates for the Junior Resident Medical appointments at the Dreadnought Hospital, Greenwich, the following have been elected for a period of six months from November 1 next: House Physicians, W. L. Crowther, M.B., B.S., F. N. H. Maidment, M.B., B.S. Lond., M.R.C.S., L.R.C.P.; House Surgeons, Alexander Porter, M.B., Ch.B. Manchester, A. H. Elmslie, M.B., Ch.B. Edin. Mr. E. Worsley Gandy, M.R.C.S., L.R.C.P., has also been appointed Honorary Anæsthetist to the Dreadnought Hospital, Greenwich.

### BOOKS RECEIVED.

George Allen and Co., Limited.

"Voice and its Natural Development." By Herbert Jennings.

Longmans, Green and Co.

"King's College Hospital." (Cooking recipes.) By Lady Ollwant, etc.

Adam and Charles Black.

"Social Guide." Mrs. H. Adams and E. A. Browne.

London Publicity Co., Ltd.

"Faith, Medicine and the Mind.' C. Reinhardt, M.D.

Spottiswoode and Co., Ltd.

"Dentists' Register."

W. Green and Sons.

"Maternity Primer." Barbour.

Charles H. Sell.

"Low's Handbook of London Charities." 1911.

John Bale, Sons and Danielsson, Ltd.

"Album of Photographs." British Medical Association.

John Wright and Sons, Ltd

"Our Baby." By Mrs. J. Langton Hewer.

The Homœopathic Publishing Co.

"The Prescriber." By John H. Clarke, M.D.

Seeley, Service and Co.

"Medical Science of To-day." By Wilmott Evans, M.D., B.Sc.

W. H. and L. Collingridge.

"Orchids for Amateurs." By C. A. Harrison, F.R.H.S.

Methodist Publishing Co.

"School Life." By T. N. Kelynack, M.D.

Cassell and Co., Ltd.

"Hygiene and Public Health." By Whitelegge and Newman.

"A Manual of Physics for Medical Students." By H. C. H. Candy, B.A., B.Sc. Lond.

"Surgical Applied Anatomy." By Treves and Keith.

"Diseases of the Skin." By Sir Malcolm Morris, K.C.V.O.

W. B. Saunders Co.

"Structure and Functions of the Body." By Fiske.

J. and A. Churchill.

"Domestic Hygiene for Nurses." By F. J. Smith.

Scientific Press.

"Sanatoria for the People." By C. H. Garland and J. D. Lister, M.D.

Hodder and Stoughton.

"Three Thousand Years of Mental Healing." By George Barton Cutten, Ph.D.

